# Active *Helicobacter pylori* Infection as a Marker of an Activity-Linked Phenotype in Chronic Spontaneous Urticaria

**DOI:** 10.14740/jocmr6511

**Published:** 2026-03-26

**Authors:** Nguyen Thi Lien, Tran Vuong The Vinh, Nguyen Thi Thanh Thanh Huyen, Le Minh Chau

**Affiliations:** a Department of Dermatology, Hai Phong University of Medicine and Pharmacy, Hai Phong, Vietnam; b Department of Surgery, Hai Phong University of Medicine and Pharmacy, Hai Phong, Vietnam; c Skinone Clinic, Ho Chi Minh City, Vietnam

**Keywords:** Chronic spontaneous urticaria, *Helicobacter pylori*, Disease activity, Clinical phenotype, Angioedema

## Abstract

**Background:**

Chronic spontaneous urticaria (CSU) exhibits heterogeneous disease activity patterns, suggesting underlying biological variability. Active *Helicobacter (H.) pylori* infection has been proposed as a potential contributor to inflammatory amplification in CSU. The aim of the study was to evaluate whether active *H. pylori* infection identifies a distinct activity-linked clinical phenotype in CSU and to examine its relationship with symptom intensity and quality-of-life impact.

**Methods:**

In the retrospective observational study, 245 adults with CSU underwent stool antigen testing for active *H. pylori* infection. Disease activity was assessed using the daily Urticaria Activity Score (UAS; range 0–6). Gradient analysis across UAS levels, phenotype feature enrichment, correlation with antigen burden, and multivariable modeling were performed. External quality-of-life impact was also evaluated.

**Results:**

Active *H. pylori* infection was detected in 64.9% of patients and was strongly associated with amplified disease activity. A graded increase in infection prevalence was observed across UAS categories, and antigen burden correlated positively with UAS (ρ = 0.540; P < 0.001). Infected patients demonstrated enrichment of severe pruritus, very high wheal counts, and angioedema. After adjustment for age, sex, and disease duration, infection remained independently associated with high disease activity (P < 0.001). Although overall quality-of-life impairment was modest, disease activity showed a weak positive correlation with external impact (P = 0.046).

**Conclusions:**

Active *H. pylori* infection may represent a marker of an activity-linked inflammatory phenotype in CSU characterized by severity gradient and phenotype clustering. While causality cannot be inferred, these findings support further prospective studies to determine whether infection-targeted strategies influence disease trajectory.

## Introduction

Chronic spontaneous urticaria (CSU) is a common inflammatory skin disorder characterized by recurrent wheals, pruritus, and, in some cases, angioedema persisting for more than 6 weeks. Although not life-threatening, CSU imposes a substantial burden on patients due to its unpredictable course, persistent symptoms, and significant impairment of quality of life. Disease activity in CSU fluctuates over time, ranging from mild, intermittent manifestations to severe, persistent disease that is difficult to control despite standard therapy [1–3].

The pathophysiology of CSU is complex and multifactorial, with mast cell activation playing a central role. While a proportion of patients exhibit autoimmune mechanisms, a large subset of CSU cases remains classified as idiopathic or CSU, in which no clear triggering factor can be identified. In recent years, increasing attention has been directed toward the role of chronic infections as potential contributors to disease activity in CSU. Among these, *Helicobacter (H.) pylori*, a gram-negative bacterium capable of inducing persistent immune activation, has been frequently implicated [2–5].

*H. pylori* infects more than half of the global population and is well recognized for its role in chronic gastritis, peptic ulcer disease, and gastric malignancy [6]. Beyond the gastrointestinal tract, *H. pylori* has been proposed as a systemic immunological trigger, capable of promoting low-grade inflammation, molecular mimicry, and dysregulated immune responses. Several studies have reported an association between *H. pylori* infection and CSU, suggesting that infected patients may experience more severe or persistent symptoms. However, existing evidence remains inconsistent, and many studies have focused primarily on treatment response following bacterial eradication rather than on the intrinsic characteristics of disease activity [7–10].

Importantly, CSU severity is increasingly conceptualized not merely as a binary state but as a structured pattern of activity encompassing pruritus intensity, wheal frequency, lesion duration, and the presence of angioedema. The Urticaria Activity Score (UAS) provides a standardized daily measure of symptom burden; however, limited data are available regarding whether active *H. pylori* infection is associated with a distinct activity-linked phenotype characterized by higher symptom intensity and specific clinical feature clustering. Furthermore, because symptom intensity is a major determinant of patient-reported burden, understanding potential quality-of-life implications alongside disease activity patterns may enhance clinical interpretation [11, 12].

Therefore, this study aimed to evaluate the prevalence of active *H. pylori* infection in adults with CSU and to examine its association with disease activity gradients, clinical phenotype enrichment, and external quality-of-life impact at baseline.

## Materials and Methods

### Study design and setting

This study was a retrospective, observational, noninterventional study conducted using routinely collected clinical data from adult patients diagnosed with CSU. Eligible patients were identified based on documented CSU diagnoses in medical records during the study period (from September 2016 to September 2024). Data were extracted from existing clinical records and laboratory databases, including demographic characteristics, disease activity assessments, clinical features, and *H. pylori* stool antigen test (SAT) results.

The study involved secondary analysis of deidentified data collected as part of routine clinical care, with no direct patient contact and no intervention or modification of standard management. All data were anonymized prior to analysis. In accordance with local regulations and institutional policies, ethical approval and informed consent were waived, as the study did not involve identifiable patient information or any procedures beyond standard care.

### Inclusion criteria

Patients were eligible for inclusion if they met all of the following criteria: 1) age ≥ 18 years; 2) a clinical diagnosis of CSU, defined as the presence of recurrent wheals, angioedema, or both, occurring daily or almost daily for a duration of more than 6 consecutive weeks, in accordance with international consensus guidelines; 3) CSU as the predominant clinical subtype, with or without associated angioedema; and 4) stable disease status at baseline, defined as no acute flare requiring systemic corticosteroids within the preceding 4 weeks.

The study population consisted exclusively of patients with CSU. Chronic inducible urticarias were excluded because they represent distinct pathophysiological entities with identifiable triggers and different disease activity patterns, which could confound the association with systemic infection.

### Exclusion criteria

Patients were excluded if they met any of the following criteria: 1) acute urticaria or urticaria with a disease duration of less than 5 weeks; 2) chronic inducible urticaria as the primary diagnosis, including but not limited to dermographism, cold urticaria, cholinergic urticaria, delayed pressure urticaria, solar urticaria, or vibratory urticaria; 3) known autoimmune diseases (e.g., systemic lupus erythematosus, autoimmune thyroid disease, vasculitis) or systemic inflammatory or infectious diseases that could confound urticaria activity; 4) urticarial vasculitis, suggested by wheals persisting longer than 24 h, residual hyperpigmentation, or histopathological confirmation; 5) active gastrointestinal disease requiring immediate medical treatment or prior gastric surgery, which could interfere with *H. pylori* assessment; 6) previous *H. pylori* eradication therapy within 6 months prior to enrollment; 7) use of antibiotics, proton pump inhibitors, bismuth compounds, systemic corticosteroids, or immunosuppressive agents within 4 weeks prior to stool sample collection, due to potential interference with *H. pylori* detection; 8) pregnancy or lactation; 9) current malignancy or history of malignancy under active treatment.

### Sample size calculation

The sample size was estimated to ensure adequate power to detect a clinically meaningful difference in disease activity between patients with and without active *H. pylori* infection. Based on prior CSU severity data, a 1-point difference in daily UAS (standard deviation (SD) of approximately 2) was considered clinically relevant. Assuming a two-sided α of 0.05 and 80% power, at least 64 patients per group were required. Given the anticipated infection prevalence and the need for multivariable logistic regression analysis, the final sample size was expanded to ensure sufficient representation in both groups and to satisfy the recommended minimum of 10 outcome events per variable included in the model. The achieved sample size of 245 patients was therefore considered adequate for the planned analyses.

### Diagnosis of CSU

The diagnosis of CSU was established based on clinical criteria, in accordance with the recommendations of the international European Academy of Allergology and Clinical Immunology (EAACI)/EU-founded network of excellence, the Global Allergy and Asthma European Network (GA^2^LEN)/EDF/World Allergy Organization (WAO) guidelines [2]. CSU was defined as the spontaneous occurrence of wheals, angioedema, or both, recurring daily or almost daily for a duration of more than 6 consecutive weeks.

All patients underwent a comprehensive clinical evaluation performed by experienced dermatologists. A detailed medical history was obtained, focusing on the onset and duration of symptoms, frequency of wheal occurrence, severity of pruritus, presence of angioedema, potential triggering factors, medication use, and personal or family history of atopic or autoimmune diseases. Physical examination was conducted to document the morphology, distribution, and transient nature of urticarial lesions.

To ensure accurate classification, patients were assessed to confirm CSU as the predominant subtype. Patients in whom inducible factors (such as physical stimuli including pressure, cold, heat, or exercise) were identified as the primary cause of wheal formation were excluded. In cases where mild inducible features were present, patients were included only if spontaneous wheals constituted the dominant clinical presentation.

Routine laboratory investigations, including complete blood count and basic inflammatory markers, were performed when clinically indicated to exclude secondary causes of urticaria. Urticarial vasculitis was excluded based on clinical features, including wheals persisting longer than 24 h, residual hyperpigmentation, pain or burning sensation, and, when necessary, histopathological findings.

### Assessment of *H. pylori* infection

Results of *H. pylori* infection were confirmed using a SAT, based on enzyme-linked immunosorbent assay. The SAT has demonstrated high sensitivity (90–95%) and specificity (90–95%) for detecting active infection in both clinical and epidemiological settings. It was selected in this study because it identifies current infection rather than prior exposure, thereby reducing misclassification bias inherent to serological testing. Furthermore, SAT is recommended by international consensus guidelines for noninvasive detection of active *H. pylori* infection. SAT was performed systematically in all eligible patients during the study period as part of routine clinical evaluation. Testing was not restricted to patients with more severe disease, thereby reducing potential selection bias.

Stool samples were collected from each patient prior to any initiation of eradication therapy and processed according to the manufacturer’s instructions. The test was interpreted qualitatively, and results were classified as positive or negative based on predefined optical density cut-off values. A positive result was considered indicative of current *H. pylori* infection.

To minimize the risk of false-negative results, patients were instructed to discontinue antibiotics, proton pump inhibitors, and bismuth-containing compounds for at least 4 weeks prior to stool sample collection. Individuals who had received *H. pylori* eradication therapy within the previous 6 months were excluded from the study [6, 7].

The SAT was selected because of its high sensitivity and specificity for active infection and its suitability for use in epidemiological and clinical studies. Based on the test results, patients were categorized into *H. pylori*–positive and *H. pylori*–negative groups for subsequent analyses. The SAT was chosen over serological assays to avoid misclassification related to past exposure rather than active infection [13–15].

### Disease activity assessment

Angioedema was identified based on documentation in medical records during the same clinical visit at which UAS was recorded. The presence or absence of angioedema was extracted from physician-documented clinical examination notes.

Disease activity was assessed using the daily UAS (range 0–6), calculated as the sum of two components: pruritus intensity (0–3) and wheal count (0–3), as recommended in international guidelines. Pruritus intensity was graded as 0 (none), 1 (mild), 2 (moderate), or 3 (severe), based on patient-reported symptom severity during the preceding 24 h. Wheal count was graded as 0 (none), 1 (< 20 wheals/24 h), 2 (20–50 wheals/24 h), or 3 (> 50 wheals/24 h), based on patient report corroborated by clinical examination when visible lesions were present. Severe pruritus was defined as a pruritus score of 3 on the UAS scale. A wheal burden of > 50 wheals/24 h corresponded to a wheal score of 3. Wheal duration > 1 h was based on patient-reported lesion persistence documented during the same clinical visit and recorded in the medical chart by the attending physician. The UAS value corresponded to the same day as SAT and was not averaged over multiple days. A UAS ≥ 5 was defined as high disease activity because it reflects moderate-to-severe daily symptom burden, corresponding to clinically meaningful activity levels in established UAS categorization frameworks.

### Quality-of-life impact assessment

The external impact of CSU on quality of life was evaluated using five items addressing social and functional domains, including: difficulty related to medication side effects, embarrassment due to urticaria symptoms, embarrassment in public settings, problems using cosmetics, and limitations in clothing choice.

Each item was scored on a 5-point Likert scale ranging from 1 (not at all) to 5 (very much), with higher scores indicating greater impairment.

### Statistical analysis

Statistical analyses were performed using SPSS 25.0. Continuous variables were expressed as mean ± standard deviation or median (interquartile range) and were compared using the Student’s *t*-test or Mann–Whitney U test. Categorical variables were expressed as frequencies and percentages and compared using the Chi-square test or Fisher’s exact test.

For descriptive gradient analysis, UAS values were categorized into individual daily scores (UAS = 4, 5, and 6). These categories reflect increasing daily symptom burden and were analyzed to evaluate whether the prevalence of *H. pylori* positivity increased progressively across higher activity levels.

To identify factors independently associated with high disease activity, multivariable logistic regression analysis was conducted. Age, sex, and CSU duration were included a priori as clinically relevant covariates, together with *H. pylori* status. Odds ratios (ORs) with 95% confidence intervals (CIs) were calculated. A two-sided P value < 0.05 was considered statistically significant.

## Results

### Infection status and activity gradient

Among 245 patients with CSU, active *H. pylori* infection was identified in 159 (64.9%). Disease activity demonstrated a structured gradient in relation to infection status. The overall prevalence of high activity (UAS ≥ 5) was 70.6%, and the probability of high activity was markedly concentrated within the infected subgroup (88.1% vs 38.4%).

A progressive increase in infection prevalence was observed across UAS categories, rising from 26.4% at UAS 4 to 67.7% at UAS 5 and 96.3% at UAS 6. In parallel, stool antigen concentration correlated positively with UAS (Spearman ρ = 0.540; P < 0.001), supporting a burden-linked activity pattern rather than a dichotomous distribution.

Baseline UAS values were higher in infected patients (5.36 ± 0.69) compared with non-infected patients (4.42 ± 0.56; P < 0.001). Median antigen concentration also differed substantially between groups (0.450 vs. 0.026 µg/mL; P < 0.001) (Table 1).

**Table 1:** Prevalence of Active *Helicobacter* (*H.*) *pylori* Infection Across the Urticaria Activity Score Gradient

UAS category	N	*H. pylori* positive, n (%)	Stool antigen, median (IQR)
UAS = 4	72	19 (26.4)	0.034 (0.018–0.123)
UAS = 5	93	63 (67.7)	0.292 (0.037–0.570)
UAS = 6	80	77 (96.3)	0.458 (0.331–0.693)

P for trend (logistic regression across UAS levels): P < 0.001. UAS: Urticaria Activity Score; IQR: interquartile range.

### Phenotype enrichment pattern

Active infection was associated with enrichment of high-intensity symptom features. Severe pruritus was documented in 81.8% of infected patients compared with 37.2% of non-infected patients (OR = 7.56; 95% CI, 4.18–13.71; P < 0.001). A pronounced clustering of very high wheal burden (> 50/24 h) was observed in infected individuals (55.3% vs. 5.8%; OR = 20.08; 95% CI, 7.72–52.22; P < 0.001). Angioedema was also more frequent in the infected subgroup (56.6% vs. 33.7%; OR = 2.56; 95% CI, 1.48–4.43; P = 0.001).

Interestingly, wheal duration > 1 h occurred less frequently among infected patients (87.4% vs 97.7%; OR = 0.17; 95% CI 0.04–0.73; P = 0.010), suggesting potential differences in lesion dynamics despite higher overall activity intensity (Table 2).

**Table 2:** Infection-Linked Phenotype Enrichment (ORs)

Clinical feature	OR (95% CI)	P value
Severe pruritus	7.56 (4.18–13.71)	< 0.001
> 50 wheals/24 h	20.08 (7.72–52.22)	< 0.001
Angioedema	2.56 (1.48–4.43)	0.001
Wheal duration > 1 h	0.17 (0.04–0.73)	0.010

OR: odds ratio; CI: confidence interval.

### Multivariable analysis for high disease activity (UAS ≥ 5)

In a multivariable logistic regression model adjusting for age (per 10 years), sex, and CSU duration (per month), *H. pylori* positivity remained strongly associated with high disease activity, with an adjusted OR of 11.84 (95% CI, 5.88–23.82; P < 0.001). Age, sex, and CSU duration were not independently associated with high activity in the adjusted model (Table 3).

**Table 3:** Multivariable Logistic Regression for High Disease Activity (UAS ≥ 5)

	Adjusted OR (95% CI)	P
*Helicobacter (H.) pylori* positive (vs. negative)	11.84 (5.88–23.82)	< 0.001
Age (per 10 years)	1.15 (0.93–1.43)	0.200
Female sex (vs. male)	1.43 (0.73–2.79)	0.300
CSU duration (per 1 month)	1.01 (0.92–1.12)	0.790

UAS: Urticaria Activity Score; OR: odds ratio; CI: confidence interval; CSU: chronic spontaneous urticaria.

### External quality-of-life impact

The overall external quality-of-life burden was modest. Among the evaluated domains, embarrassment related to urticaria symptoms demonstrated the highest mean score (2.05 ± 0.42), whereas other domains remained closer to the lower end of the scale.

Total external impact scores did not differ significantly according to infection status (7.75 ± 1.39 vs. 7.54 ± 1.32; P = 0.30). However, disease activity itself correlated weakly but significantly with overall quality-of-life impairment (Spearman ρ = 0.13; P = 0.046).

Patients with active infection reported greater difficulty related to medication side effects (1.40 ± 0.56 vs. 1.08 ± 0.28; P < 0.001), whereas other domains showed no statistically significant differences (Table 4).

**Table 4:** External Quality-of-Life Impact According to *H. pylori* Status

	*H. pylori*–	*H. pylori*+	P value	Spearman ρ with UAS	P value
Difficulty due to medication side effects	1.08 ± 0.28	1.40 ± 0.56	< 0.001	0.28	< 0.001
Embarrassment due to urticaria symptoms	2.12 ± 0.45	2.02 ± 0.40	0.08	–0.04	0.52
Embarrassment in public places	1.42 ± 0.54	1.48 ± 0.62	0.60	0.01	0.86
Problems using cosmetics	1.33 ± 0.47	1.37 ± 0.52	0.68	0.05	0.49
Limitation in clothing choice	1.59 ± 0.54	1.48 ± 0.53	0.13	0.07	0.31
Total score (5–25)	7.54 ± 1.32	7.75 ± 1.39	0.30	0.13	0.046

Data were presented as mean ± standard deviation. *H. pylori*: *Helicobacter pylori*; UAS: Urticaria Activity Score.

## Discussion

This study demonstrates that *H. pylori* positivity was common among patients with CSU and was strongly associated with higher disease activity, enrichment of severe clinical features, and a measurable gradient across UAS levels. In addition, although the overall external quality-of-life impact was modest, disease activity correlated weakly but significantly with patient-reported impairment. These findings suggest that *H. pylori* infection may characterize a biologically and clinically distinct subgroup of CSU with amplified inflammatory expression.

*H. pylori* infection may represent an associated factor linked to higher disease activity in a subset of patients.

Multiple meta-analyses over the past decade have reported a significant association between *H. pylori* infection and CSU and notable heterogeneity attributable to differences in diagnostic tests, regional prevalence, and case definitions [9–11]. The study contributes a larger effect size for severe activity (UAS ≥ 5) and, critically, adds evidence of a dose–response-like relationship between infection burden (stool antigen levels) and disease activity, an aspect that is often absent from cross-sectional case–control literature.

The prevalence of *H. pylori* positivity (64.9%) appears higher than rates reported in some regional studies, where infection prevalence among CSU cases may range widely depending on geography and testing modality. This discrepancy likely reflects differences in background prevalence and, importantly, the use of assays for active infection rather than serology. Prior cross-sectional studies using SAT have similarly emphasized the practicality of detecting active infection, though absolute positivity rates vary [13, 14, 16, 17].

The data highlighted a distinctive phenotype enrichment in *H. pylori*–positive CSU: severe pruritus, very high wheal counts (> 50/24 h), and more frequent angioedema. The angioedema signal is clinically meaningful because the presence of angioedema often correlates with higher disease burden and therapeutic complexity. International urticaria guidelines recognize CSU as a mast cell–driven disease presenting with wheals, angioedema, or both, and recommend targeted diagnostic workup rather than indiscriminate testing. The findings suggest that testing for active *H. pylori* infection may be particularly informative in patients with high disease activity and an angioedema-prone phenotype [1–4].

Previous studies have suggested that antibiotic regimens aimed at *H. pylori* eradication can improve CSU outcomes in some patients, although the strength and mechanisms remain debated. A placebo-controlled double-blind trial reported symptomatic benefit of eradication as add-on therapy, albeit described as temporary. More recently, systematic reviews and meta-analyses of randomized trials have reported improved remission rates with eradication antibiotics compared with control, though trial sizes are modest and between-study heterogeneity exists. Observational analyses using methods such as propensity score matching have also suggested clinical benefit from eradication strategies in CSU [18, 19].

Mechanistically, *H. pylori* is known to drive chronic immune activation and has been implicated in various extra-gastric inflammatory manifestations. Proposed pathways relevant to CSU include immune dysregulation, molecular mimicry, and enhanced mast cell priming. Reviews on *H. pylori* virulence determinants, such as CagA/VacA-related inflammatory signaling, support biological plausibility for systemic effects beyond the stomach, although direct mechanistic links in CSU remain to be confirmed. The dose–response-like findings (higher antigen burden associated with higher UAS) are compatible with an “infection burden → inflammatory priming → amplified mast cell reactivity” model, but mechanistic endpoints were not assessed in this study [20–22].

The magnitude of the adjusted odds ratio (about 12) is substantial for an observational analysis. Such effect sizes may reflect phenotype enrichment within the infected subgroup but also raise the possibility of residual confounding. Unmeasured variables—including metabolic factors, atopic predisposition, and lifestyle determinants—may have contributed to the observed association. Accordingly, the findings should be interpreted as demonstrating a strong association rather than a causal relationship.

The quality-of-life findings provide important context. While *H. pylori*–positive patients did not exhibit substantially higher composite external impact scores, disease activity itself was weakly associated with impairment. This pattern suggests that infection may influence symptom intensity more strongly than psychosocial burden. The highest impairment domain was embarrassment related to urticaria symptoms, consistent with previous literature emphasizing the visible and stigmatizing nature of wheals. Although active infection was strongly associated with symptom intensity, it did not translate into substantially higher composite quality-of-life scores. This suggests that infection may amplify clinical activity more prominently than psychosocial burden.

The study showed that wheal duration > 1 h was less frequent among *H. pylori*–positive patients despite their markedly higher activity. One plausible explanation is a difference in wheal dynamics: *H. pylori*–positive patients may experience frequent, high-count wheals that resolve within shorter intervals but recur continuously, yielding higher overall UAS. Alternatively, patient-reported duration may be subject to measurement error. This signal should be interpreted cautiously and may warrant objective diary-based measurement in future studies.

The present findings support a more phenotype-driven approach to diagnostic evaluation in CSU. In patients presenting with high disease activity, severe pruritus, very high wheal burden, or concomitant angioedema, assessment for active *H. pylori* infection may be considered as part of a targeted workup. Such an approach may help identify a subgroup characterized by amplified inflammatory expression and potentially modifiable contributing factors. In Vietnam, eradication therapy is recommended when active *H. pylori* infection is confirmed, in accordance with international consensus guidelines. Nevertheless, routine screening of all CSU patients is not universally practiced and remains clinician-dependent. The data suggest that selective testing in patients with moderate-to-severe activity may represent a pragmatic strategy, although prospective interventional studies are required to determine whether this approach translates into improved clinical outcomes.

This study had several limitations. The retrospective cross-sectional design precludes assessment of temporal relationships and does not allow causal inference. *H. pylori* status was determined using SAT, which identifies active infection but does not provide information on bacterial virulence, chronicity, or host–pathogen interactions. Immunologic biomarkers were not measured, limiting mechanistic interpretation. Important potential confounders, including body mass index, smoking, metabolic comorbidities, and atopic history, were not consistently available and could not be included in multivariable analyses, leaving residual confounding possible. Validated patient-reported outcome measures recommended in current urticaria guidelines, such as the Urticaria Control Test (UCT) and Angioedema Control Test (AECT), were not available in this retrospective dataset, limiting the evaluation of disease control and patient-reported burden. The single-center design and regional infection prevalence may limit generalizability. Additionally, only baseline disease activity was analyzed, without evaluation of longitudinal outcomes or treatment response. Prospective, multicenter, and mechanistic studies are needed to clarify causal relationships and clinical implications.

### Conclusions

*H. pylori* infection was independently associated with higher disease activity and enrichment of severe clinical features in patients with CSU. A clear severity gradient was observed across UAS levels, and infection burden correlated with symptom intensity. Although overall external quality-of-life impairment was modest, higher disease activity was associated with greater patient-reported impact. These findings identify a clinically distinct CSU phenotype linked to active *H. pylori* infection. Given the retrospective cross-sectional design, causality cannot be inferred. Prospective longitudinal and interventional studies are needed to clarify whether targeted *H. pylori* evaluation and eradication may influence disease outcomes.

## Data Availability

The data supporting the findings of this study are available from the corresponding author upon reasonable request.

## Abbreviations

CI: confidence interval
CSU: chronic spontaneous urticaria
OR: odds ratio
SAT: stool antigen test
SD: standard deviation
UAS: Urticaria Activity Score
